# Three-dimensional shape from shading is modulated by top-down attention: Evidence from event-related potentials

**DOI:** 10.1177/20416695251350000

**Published:** 2025-07-13

**Authors:** Joshua P. Matthews, Debra L. Mills, Ayelet Sapir

**Affiliations:** 1 1506 Bangor University, UK; 2 4918 University of Greenwich, UK This paper has also been awarded the Early Career Best Paper Prize.

**Keywords:** three-dimensional shape, depth perception, shape from shading, top-down attention, event-related potentials, lateralisation

## Abstract

Shading is an important monocular cue for three-dimensional (3D) perception, whereby 3D shape can be inferred from shading patterns across an object, in a process termed *shape-from-shading*. Shape-from-shading has been characterised as a pre-attentive process that occurs in parallel across the visual field. Recent evidence, however, has challenged this notion, suggesting that it consists of an early pre-attentive process and a later stage of processing that is reliant on top-down attention. Here, we use event-related potentials (ERPs) to test this claim whilst participants were instructed either to ignore or to attend to shaded stimuli that could be perceived as two-dimensional (2D) and 3D. We found that 3D stimuli evoked a larger N1 component than 2D stimuli in both attended and unattended conditions, implying an early, pre-attentive processing stage in shape-from-shading. This activity was lateralised to the right hemisphere when participants attended to the stimuli, in accordance with the right hemisphere advantage in top-down attention. Further, when participants attended to the stimuli, a larger N2 component for 3D compared to 2D shape was found, suggesting a late, top-down process for identifying 3D shape. These findings provide evidence for two distinct stages of processing for shape-from-shading and suggest that attention is necessary for the perception of shape-from-shading.

## How to cite this article

Matthews, J. P., Mills D. L., & Sapir, A. (2025). Three-dimensional shape from shading is modulated by top-down attention: Evidence from event-related potentials. *i-Perception*, 16(4), 1–30. https://doi.org/10.1177/20416695251350000

Three-dimensional (3D) perception is an aspect of human vision that is fundamental for our ability to interact with objects in our environment (Durand et al., 2009). Without 3D perception, an individual may find themselves unable to perform simple tasks such as opening a door or identifying the edge of a cliff (Orban, 2011). Given that retinal input is two-dimensional (2D), a key area of research has been to explore how the visual system utilises this information to create the perception of a 3D environment (Finlayson et al., 2017). Several visual cues have been found to facilitate this process. This includes binocular cues such as binocular disparity and binocular convergence; as well as monocular cues such as texture, motion parallax and shading (Welchman, 2016). Of particular interest here is the monocular cue of shading, which gives rise to 3D perceptions of objects through depth information carried by the shading pattern across an object, combined with the direction of the light source. This process is typically referred to as ‘shape from shading’ (Kleffner & Ramachandran, 1992).

Early research has suggested that shape from shading is processed in a pre-attentive manner (that is, rapidly and without the need for attention, e.g., Treisman, 1985) and in parallel across the visual field (Braun, 1993; Enns & Rensink, 1990; Enns & Rensink, 1991; Kleffner & Ramachandran, 1992; Sun & Perona, 1996). For instance, Sun and Perona (1996) presented participants with a series of feature search tasks, comparing performance in search arrays where the target and distractors were either 3D cubes created through shape from shading, or 2D objects such as line patterns and line cubes. In each one of the displays, participants were required to find a target object, for instance in 3D arrays the target stimuli were the same cube used as the distractor stimuli but rotated 180°. The number of distractor cubes in the 3D array did not significantly influence reaction times for target cubes, but directly increased the response times in the 2D arrays, suggesting that attention was needed to find 2D targets amongst 2D distractors, but not for 3D targets amongst 3D distractors. Moreover, Braun (1993) found that when multiple shapes from shading objects appeared in different positions in an array, the participants could identify the presence of both simultaneously, suggesting that identification of shape from shading occurs in parallel across the visual field. Participants were also presented with a word discrimination task, with task-irrelevant shape from shading stimuli appearing in various parts of the display. Despite participants attending to the letter discrimination task, the 3D effect created by the shape from shading stimuli was still reported even though these stimuli were unattended. As a result, early research proposed that shape from shading appears to be processed without the need for attention, suggesting that shape from shading is completed at an early stage in visual processing.

The light from above prior, a component of shape from shading, refers to the default assumption that the light source for the object is located above and slightly to the left of the object (Kleffner & Ramachandran, 1992; Mamassian & Goutcher, 2001; Pickard-Jones et al., 2020; Sun & Perona, 1998), with some suggesting that this left bias may be as a result of a right hemispheric dominance in visual processing (e.g., Mamassian & Goutcher, 2001). Studies investigating the light from above prior have also suggested that shape from shading occurs early in visual processing. Early research has investigated whether this assumed light source is computed using either gravitational (e.g., using environmental cues) or retinal frames of reference (Howard et al., 1990; Kleffner & Ramachandran, 1992). Kleffner and Ramachandran (1992) presented an array of convex and concave stimuli to participants and asked participants if the convex stimuli in the display constructed an unbroken circle (e.g., ‘O’) or a broken circle (e.g., ‘C’). In half of the stimulus presentations, participants sat upright in front of the display, whilst for the other half of the stimulus presentations participants viewed the stimulus display with their head angled at 90° from the display. They found that the light from above prior was computed in regard to retinal, rather than gravitational frames of reference, as tilting the participant's head altered their perception of the stimuli array. Such evidence has been used to suggest that shape from shading is computed early in the visual system, as the assumed light source direction for shaded objects is not adjusted to account for environmental cues. Indeed, evidence from Mamassian et al. (2003), using event-related potentials (ERPs), provided further support for the notion that the light from above prior is calculated early in the visual system. They tested participants while viewing shape from shading stimuli and found that activity around 100ms post stimulus onset in occipital areas corresponded to the left bias in the assumed light source direction for shaded objects. As this activity was found early in processing and over visual areas, the authors argued that this further supports the notion that shape from shading operates in a bottom-up manner without the support of higher cortical areas.

Despite evidence that shape from shading is processed early in the visual system, and without the need for attention, more recent evidence has challenged this notion. The early and pre-attentive account of shape from shading relied heavily on evidence from feature search tasks (e.g., Kleffner & Ramachandran, 1992; Sun & Perona, 1996). However, the notions put forward by the Reverse Hierarchy Theory (RHT) suggest that activity in higher-level cortical areas, along with the spreading of attention, is necessary for the pop-out effects in feature search tasks (Ahissar & Hochstein; 2004; Hochstein & Ahissar, 2002), such as that found for shape from shading. Two modes of vision are identified in this model: an initial generalised visual mode which provides a rapid account of the visual scene and is used to guide a latter visual mode that operates on scrutinising specific details of the object. When applied to shape from shading, it is possible that some information on shape from shading is acquired rapidly, and this is used to guide attention to these objects for further processing related to 3D shape. Indeed, there is research that supports the notion that higher-level cortical areas are implicated in pop-out effects during feature search tasks. Hershler and Hochstein (2005) demonstrated that faces could produce a pop-out affect, like shaded spheres, and that this pop-out affect with faces was mediated by holistic face processing, a well-studied higher-level aspect of face perception (Pellicano & Rhodes, 2003; Wang et al., 2012; Towler & Eimer, 2013). The finding that holistic processing was responsible for producing a pop-out affect strengthens the notion that higher-level cortical areas are implicated in feature search tasks. Therefore, the presence of a pop-out affect in search tasks is not sufficient to argue that a property is processed pre-attentively and only requires low-level processing. Consequently, it is possible that there is an element of processing for shape from shading that requires attentional processes.

The claim that shape from shading is completed early in visual processing, demonstrated by a retinotopic frame of reference for the light from above prior, has also faced contradictory evidence. Some research has since shown that gravitational frames of reference can be adapted by the light from above prior (Adams, 2008; Barnett-Cowan et al., 2018; Jenkin et al., 2004). Adams (2008) compared the effects of head tilt in two separate shape from shading tasks. In the first task, participants were asked to identify the 3D shape of a shaded stimulus (e.g., convex or concave), whilst in the second task participants were asked to make a response as to whether a shaded target stimulus was present amongst an array of shaded distractors. Both tasks were completed with the participants’ head fixed at −60°, 0° or 60° from the display. They found that the gravitational frame of reference influenced the assumed light source position when participants were asked to make judgements about the shape of the object, but a retinal frame of reference was clearly dominant during their visual search task. This suggests that different strategies are employed by the visual system dependant on the task demands, with rapid processing employed whilst searching for objects, but with additional processing required when judging the 3D shape of an object.

Another finding that challenges the notion that shape from shading is processed early in the visual system comes from evidence that shape from shading has been shown to engage higher-level cortical areas (Georgieva et al., 2003, 2008; Gerardin et al., 2010; Gillebert et al., 2015; Sereno et al., 2002; Taira et al., 2001). Taira et al., (2001) utilised functional magnetic resonance imaging (fMRI) whilst participants either decided if a shaded stimulus was convex or concave or reported a colour inside of the stimuli. It was found that the intra-parietal sulcus (IPS) showed greater activation when participants indicated the shape of the shaded stimuli, compared to when they reported the colour inside the stimulus. Interestingly, this activity for shape from shading was lateralised to the right IPS, suggesting a dominance of the right hemisphere in processing shape from shading. Such a hemispheric dominance in 3D shape processing has also been supported by work demonstrating a right lateralisation of early processing related to depth processing (Gao et al., 2015; Séverac Cauquil et al., 2006), as well as the global processing of 3D shapes (Leek et al., 2016). Furthermore, the involvement of the IPS in shape from shading was also found by Gerardin et al. (2010). Here, the authors investigated the neural activity associated with ‘light’ and ‘shape’ processing of shape from shading. Participants were presented with a ring that contained eight segments along its outer edges, and the lighting direction of the stimulus was manipulated to produce convex and concave perceptions of each segment of the stimuli. Whilst seven of the eight segments shared the same shape in all lighting conditions, one segment was perceived as the opposite shape of the rest of the segments within the stimuli. Participants were required to report the shape of this ‘odd’ segment of the stimuli (concave or convex). It was revealed that light processing was associated with activity in early occipital areas, but 3D shape processing was instead associated with activity in higher areas, specifically in IPS. There is some conflicting evidence to this notion however, with other research suggesting that ventral visual areas such as the caudal inferior-temporal gyrus (ITG) are instead vital to perceiving shape from shading (Georgieva et al., 2008), although such a difference may be due to differences in task demands. Regardless, such research suggests that the identification of the 3D shape of an object is reliant on the involvement of higher-level cortical areas outside of early visual areas such as V1, indicating that the processing of 3D shape from shading is not completed solely by early visual areas. Furthermore, the findings of Taira et al. (2001) also show that the involvement of higher-level cortical areas in shape from shading may require attention to the object, as the activation of the IPS was only found when attention was guided to the stimulus shape. Finally, these studies also suggest the involvement of the right hemisphere in shape from shading, as the IPS activation was lateralised to the right hemisphere (Taira et al., 2001).

Interestingly, there is recent evidence to suggest that identifying the 3D shape of an object requires top-down (guided) attention. The ‘darker is deeper’ heuristic refers to an assumption whereby individuals assume that darker surfaces are more likely to be seen as deep (Langer & Zucker, 1994). Sapir et al. (2021) used pupillary responses to assess if perceiving surfaces as deeper leads to the assumption they are also darker. They presented participants with shaded objects that could be perceived as either convex or concave dependant on their orientation and found that the illusions of concavity elicit pupil dilations and illusions of convexity elicit pupil constrictions. This was only found when participants judged the shape of the stimuli or were made aware of the depth in the stimuli, but not when participants viewed the stimuli passively. This suggests that participants did not perceive shape from shading whilst attention was not guided to the stimuli, and implies that top-down attentional processes are required to identify shape from shading, contradictory to bottom-up and pre-attentive accounts of shape from shading. The authors proposed that shape from shading consists of two distinct stages: an early pre-attentive processing stage where the object is segregated from the background, and a later shape identification stage that is reliant on top-down attentional processes to perceive the 3D shape of a shaded object.

The current study aimed to investigate whether top-down attention is required for a later stage of processing related to shape from shading. We hypothesise that shape from shading involves not only an early stage of processing of depth, but also a later stage that identifies the 3D shape of the object and requires top-down attention. To test this, we used ERPs as they provide excellent temporal resolution of sensory and cognitive processing (Luck, 2014), and therefore have the potential to distinguish between early and late stages of processing for shape from shading. ERP studies identified early (P1 and N1) and relatively later (P2 and N2) components that appear to be sensitive to the processing of 3D objects (Gao et al., 2015; Hou et al., 2006; Kasai & Morotomi, 2001; Mamassian et al., 2003; Omoto et al., 2010; Pegna et al., 2018; Omoto et al., 2012; Séverac Cauquil et al., 2006). Specifically, early processing for depth perception has been suggested to occur during the time range of the P1 and N1 components (e.g., Gao et al., 2015; Omoto et al., 2010; Séverac Cauquil et al., 2006), whilst processing for 3D shape has been suggested to occur later in processing, during the time period of the P2 and N2 components (e.g., Gao et al., 2015; Mamassian et al., 2003; Omoto et al., 2010; Pegna et al., 2018). As such, we predict that shape from shading stimuli will evoke a larger P1 and N1 component than 2D stimuli, providing evidence for early processing related to the identification of depth from shading. We also predict that shape from shading stimuli will evoke a larger P2 and N2 component than 2D stimuli, only when attention is guided to the stimuli, reflecting the later stage of processing for shape from shading that is dependent on the deployment of top-down attention.

## Methodology

### Power Analysis and Pre-Registration

Power analysis was conducted using G*Power (Faul et al., 2007), which suggested that a minimum of 34 participants were required to detect a medium effect size at 80% power for our planned analyses. We powered the experiment to detect a medium effect size based on the size of the differences observed in each component for each shape during a pilot study conducted prior to the main experiment. This pilot study was conducted with 11 participants. As ERPs vary widely across different paradigms (Jensen & MacDonald, 2023), we used a pilot study with the same paradigm as the current experiment to estimate an expected effect size in the current study. The study was pre-registered at aspredicted.org (https://aspredicted.org/5jn2-gqw8.pdf).

### Participants

Forty-five participants were recruited for the experiment, all of which were psychology students at Bangor University. We recruited more participants than required to account for participants that needed to be excluded from analyses. The cohort was aged between 18 and 29 years old (*M* = 20.53, *SD* = 3.00), and consisted of 29 females, 12 males and 4 participants who did not conform to traditional gender norms. All participants were recruited using the Psychology participation pool and possessed normal or corrected-to-normal vision. Overall, 35.66% of the cohort were bilingual and 80.00% of participants were right-handed. Thirty-seven of the participants reported having no neuropsychological conditions, whilst others in the cohort reported having dyslexia, autism and attention deficit hyperactivity disorder (ADHD). In accordance with the guidelines of our ethics approval, we did not prevent any participants from participating in the experiment on the basis of demographic information. Fully informed consent was obtained from all participants prior to testing, and all participants were fully debriefed from the study after completing the experiment. This experiment was approved by the Bangor University ethics committee for the School of Psychology and Sport Science (ethics application number: 2023–17317) prior to any data being collected, and participants were awarded with course credits for their participation.

### Task Apparatus

The experiment was created using Presentation^®^ software (Version 23.0, Neurobehavioral Systems, Inc., Berkeley, CA, www.neurobs.com) and presented on a Samsung flat screen monitor, with a refresh rate of 60Hz and a resolution of 1920×1080 pixels. The Biosemi ActiView program (https://www.biosemi.com/software_biosemi_acquisition.htm) was utilised for EEG acquisition. Participants responded to the experiment with a three-button response box, containing 2 black buttons on the top of the device and 1 red button on the bottom of the device.

### Design

The current experiment asked participants to either ignore or attend to 2D and 3D objects that appeared before a colour search task whilst we recorded ERPs. Therefore, the study consisted of a 2×2 Within-Subjects design, with the factor of shape containing two levels (3D / 2D) and attention containing two levels (unattended/attended). 3D objects would appear as either convex or concave. The dependant variables of interest were the amplitude of the P1, N1, P2 and N2 components.

### Stimuli

**Figure 1.**
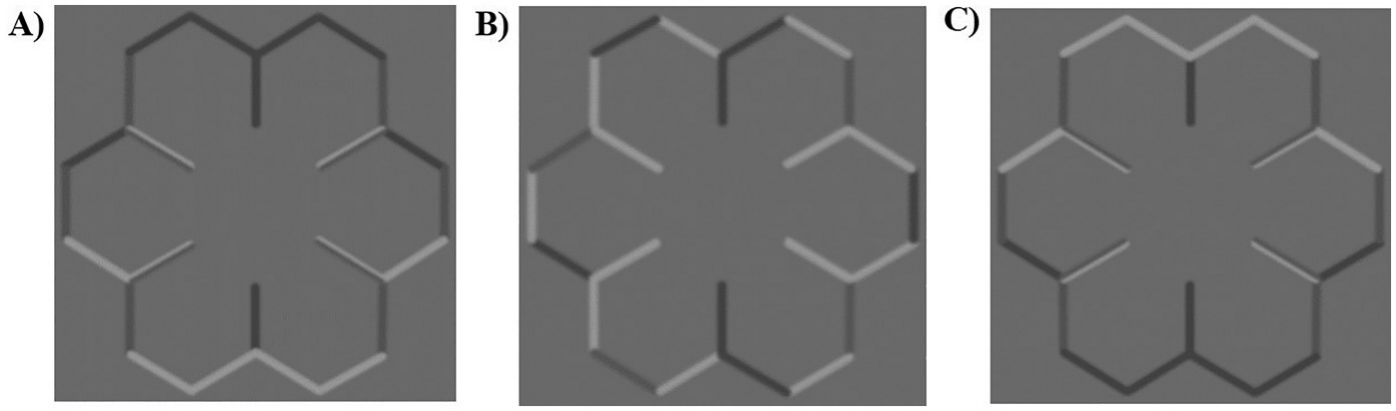
2D and 3D stimuli used in the experiment. (A) The 3D snowflake stimulus, presented at 180° orientation, creating the perception of concavity. (B) The 2D snowflake stimulus, perceived as neither convex nor concave, but as an ambiguous 2D object. (C) The 3D snowflake stimulus, presented at 0° orientation, creating the perception of convexity.

All stimuli and text in the experiment were presented on a grey background (RGB: 128, 128, 128). The 3D and 2D stimuli are shown in Figure 1. The 3D snowflake stimulus was used to create 3D perceptions of convex and concave shape from shading (Sapir et al., 2021). This stimulus utilises the light from above prior to create the impression of 3D shape. Specifically, the bright edges of the stimuli were positioned at the top of the stimulus when presented at an orientation of 0°, creating the perception of convexity, whilst the bright edges were positioned at the bottom of the stimulus at an orientation of 180°, creating the perception of concavity. The 3D snowflake stimulus was presented at both 0° and 180° orientations during this experiment. A control stimulus in the form of a 2D snowflake stimulus was created using alternating dark and bright lines. Therefore, the light across the object was inconsistent with any pattern of shading that would produce the perception of 3D shape and so this object appeared as an ambiguous 2D object^1^. It is important to note that all stimuli in the current experiment were physically 2D, but the perception of 3D shape here is created through illusionary mechanisms caused by the shading patterns across the objects. The term 2D here is used to describe the stimulus that does not evoke a stable perception of 3D shape but rather appears ambiguous in shape or flat. Both the 3D and 2D stimuli consisted of dark grey (RGB: 82, 82, 82), medium grey (RGB: 100, 100, 100) and light grey (RGB: 166, 166, 166) lines. A Gaussian blur of 1.4 pixels was applied to both stimuli. The 3D and 2D stimuli subtended 10° of the participants’ visual field, with participants seated 120cm away from the monitor.

For the colour search task, yellow (RGB: 255, 255, 0), red (RGB: 255, 0, 0) and pink (RGB: 255, 192, 203) boxes were created. Eight boxes were shown at a time, with 4 in the left visual field and 4 in the right visual field. Each box subtended a visual angle of 1.5°, placed 3° away from one another both vertically and horizontally. The maximum distance from the centre of the screen the boxes could appear were −12°/ 12° on the *x* axis, and −15°/15° on the *y* axis, and the possible location of boxes along these axes varied in increments of 3°.

### Procedure

Informed consent was obtained from participants prior to the study commencing. Participants provided demographic information before being seated in a faraday cage, and all electronic devices were removed from the participant. Once the EEG set up procedure was complete, participants were instructed to limit their blinking and movement as much as possible during the experiment. Participants were told they would be presented with a colour search task, whereby they would view an array of coloured boxes on the screen. Participants were instructed to press the top left button if a red box was present in the array, and to press the top right button if no red box was present. Participants were also informed at the beginning of the experiment that some objects would appear before this colour search task, which were not part of the task and did not require a response.

**Figure 2.**
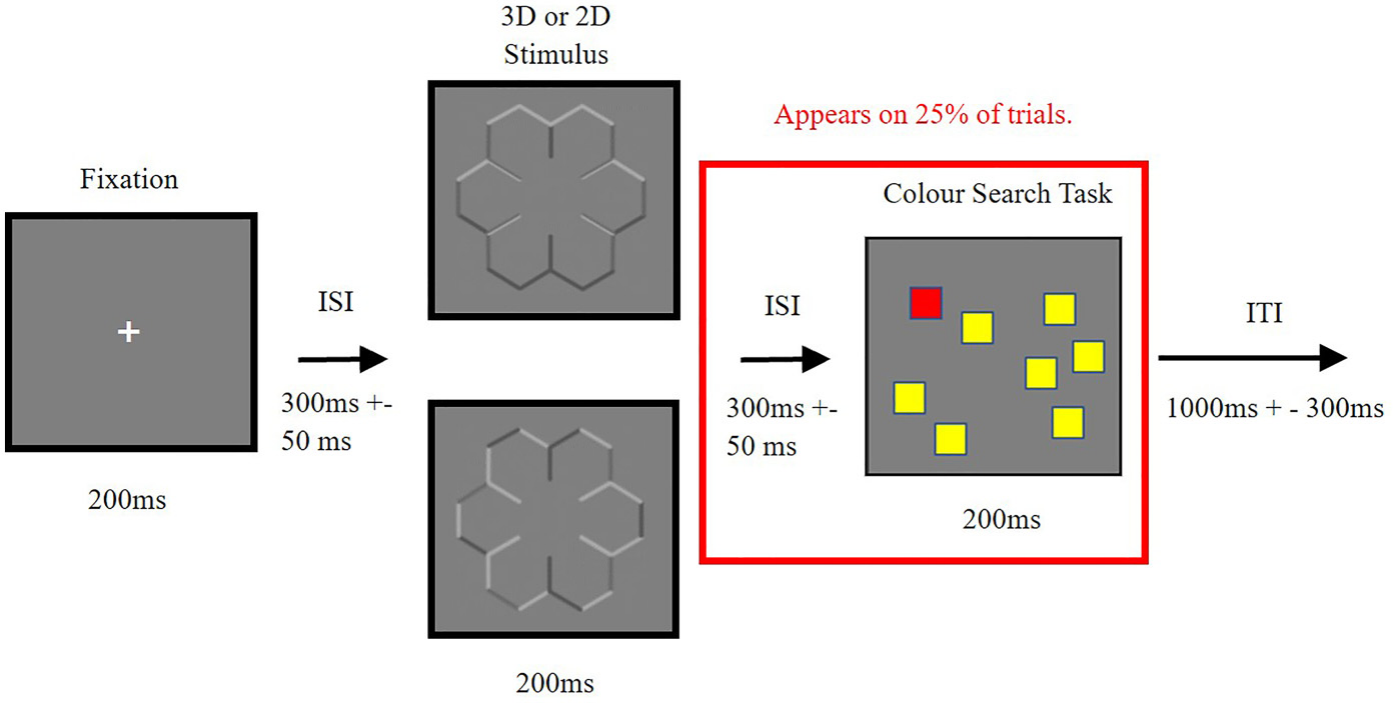
Demonstrating the procedure for trials during the experiment. Participants are presented with the fixation cross at the centre of the screen, followed by a 3D or a 2D snowflake stimulus, and then on 25% of trials participants are also presented with a colour search task, requiring a response from the participant. ms = milliseconds, ISI = Inter-stimulus interval, ITI = Inter-trial interval.

The procedure of an experimental trial is shown in Figure 2. Participants were first presented with a 200 ms white fixation cross, followed by an inter-stimulus interval of 300 ms (+–50 ms). They were then presented with either the concave, 2D or convex stimulus for another 200 ms^2^. An inter-trial interval was then presented for 1000 ms (+–300 ms), and the next trial then began. On 25% of the trials, following the 3D or 2D stimulus, there was another inter-stimulus interval of 300 ms (+–50 ms), followed by a colour search task for 200 ms. Participants responded once the coloured boxes disappeared, and the inter-trial interval was shown before the next trial was presented. In the colour search task, 50% of trials contained no target box or pink box, 25% contained the target box (the red box) and 25% contained a pink box. This pink oddball box was included to aid in maintaining participant engagement throughout the study.

Participants were first presented with 16 practice trials of the experiment. After this, participants were reminded of the task and to passively view the stimuli proceeding the search task. They were then presented with the experimental trials for the unattended viewing condition, consisting of 8 blocks of 40 trials, with 320 trials in total. Of these 320 trials, 80 contained the colour search task, with 40 trials containing no target, 20 containing a target box and another 20 containing the pink non-target oddball box. After each block, participants were given an opportunity to take a break before continuing to the next block. After eight blocks of the unattended condition, participants were presented examples of the convex, concave and 2D stimulus, and told that these examples should appear to ‘pop-out’ (convex), be ‘pushed-in’ (concave) and be flat, respectively. They were then instructed to attend to the stimuli that appear before the colour search task and were told that they may be asked a series of questions about these stimuli after the experiment. A further eight blocks of the experiment during this attended viewing condition were then presented to participants in an identical manner to the unattended viewing condition, but with each resting screen between blocks now reminding participants to attend to the stimuli proceeding the colour search task. The order of the unattended and attended blocks was not counterbalanced during the study, as demonstrating the presence of the 3D shape in the stimuli before the unattended viewing condition may have hindered participants’ ability to ignore these stimuli. After the attended viewing blocks, participants were shown each orientation of the 3D stimulus, along with the 2D stimulus, and they were asked if they perceived each stimulus as appearing to ‘pop-out’ (convex), be ‘pushed-in’ (concave) or flat. The EEG equipment was then removed from the participant, and they were fully debriefed from the experiment.

### EEG Acquisition and Pre-Processing

EEG was recorded using the 64-channel Biosemi Active-Two system (Biosemi B.V., Amsterdam, Netherlands), with electrodes placed into a Biosemi elastic cap in accordance with the extended 10/20 system (Left Electrodes: Fp1, AF3, AF7, F1, F3, F5, F7, FC1, FC3, FC5, FC7, C1, C3, C5, T7, CP1, CP3, CP5, TP7, P1, P3, P5, P7, PO3, PO7, P9 & O1. Right Electrodes; Fp2, AF4, AF8, F2, F4, F6, F8, FC2, FC4, FC6, FC8, C2, C4, C6, T8, CP2, CP4, CP6, TP8, P2, P4, P6, P8, PO4, PO8, P10 & O2. Midline Electrodes: Fpz, Afz, Fz, FCz, Cz, CPz, Pz, POz, Oz & Iz). The driven right leg (DRL) and common sense mode (CMS) electrodes were also used, with these electrodes acting as the ground electrodes. The EEG was recorded filter and reference free, and a flat electrode was placed on the left and right mastoids. Eye blinks were measured by placing a flat electrode (EOG1) under the participant's left eye, and a horizontal electrooculogram (HEOG) was recorded by placing a flat electrode to the side of the participant's right lateral canthus. Each set of 32 active electrodes was connected to an A/C recorder, along with the DRL/CMS and flat electrodes. Before recording EEG data, the offset of all electrodes was reduced to at least 25 µV and kept under 30 µV whilst recording, relating to the impedance of the electrodes in the Biosemi system. EEG was recorded with a sampling rate of 2048 Hz and after recording, data were decimated to a sampling rate of 512 Hz.

For pre-processing, we used the ERPLAB toolbox (Lopez-Calderon & Luck, 2014), running under EEGLAB (Delorme & Makeig, 2004) in MATLAB. After importing the participant's data, we filtered the EEG recording with a digital bandpass filter of 0.1–30 Hz, using a roll-off slope of 12 dB/octave and utilising the IIR Butterworth filter. Following this, the data were re-referenced to the average signal of the flat electrodes placed on the left and right mastoids (Luck, 2014). The vertical electrooculogram (VEOG) channel was also created by subtracting the signal of electrode Fp1 by the signal of electrode EOG1. Epochs of −200 to 1500 ms were created, with 0ms being the onset of the event of interest. We then used artefact rejection to reject epochs containing artefacts between −200 and 800 ms post-stimulus onset, using automatic artefact rejection with a window-to-window threshold criterion of 100 µV. Epochs were then checked through manual inspection to ensure that epochs containing artefacts were correctly rejected. Average ERPs were then created from the participant's data and were time-shifted backwards by 40 ms to match the delay between stimulus onset and the associated event-code marker. Once this was conducted for all participants, we then averaged all average ERPs across all participants to create grand-average ERPs for the conditions of interest.

To identify the distribution and time window for each component, a collapsed localiser (Keil et al., 2014; Luck & Gaspelin, 2017) was created by averaging the ERPs gathered from all shapes over all conditions. This was further assessed by plotting 5 ms epochs on topographical maps to observe the onset and offset of each component. The time window specified for each component, along with electrodes representing the distribution of the component, were as follows:

***P1***: 105–145ms. Left electrodes: O1, PO7, PO3, P3, P5 & P7. Right electrodes: O2, PO8, PO4, P4, P6 & P8.

***N1***: 150–200ms. Left electrodes: O1, PO3, PO7, P3, P5, P7 & P9. Right electrodes: O2, PO4, PO8, P4, P6, P8 & P10.

***P2***: 215–285ms. Left electrodes: O1, PO3, PO7, P1, P3, P5 & P7. Right electrodes: O2, PO4, PO8, P2, P4, P6 & P8.

***N2***: 290–350ms. Left electrodes: O1, PO7 & PO3. Right electrodes: O2, PO8 & PO4.

### Data Analysis

We conducted analyses using the average amplitude of all electrodes over the left and right hemisphere where a given component was observed to reduce type 1 errors produced by comparing multiple electrodes or regions in analysis (Luck, 2014). We also assessed if there was an asymmetry in the distribution of a given component, where the amplitude of a component may be larger over one hemisphere than the other. Further, we investigated any effects of laterality, whereby the magnitude of an experimental effect in one hemisphere may be larger than that in the other hemisphere. In other words, we assessed whether the size of a difference between conditions is larger in one hemisphere than another. Participants were excluded from analysis if there were fewer than 50 artefact-free trials for 2D and 3D stimuli in each attentional condition. However, as we observed clear differences between convex and concave objects during visual inspection of the data, we conducted analyses on concave, convex and 2D shapes separately, rather than the planned analysis of 3D (average of convex and concave) compared to 2D shape. Therefore, we also required that each participant obtained at least 30 artefact-free trials for convex and concave objects in each viewing condition, as this has been found to be the minimum number of trials to achieve high internal consistency when measuring ERPs (Thigpen et al., 2017). Following artefact rejection, the average number of trials for each shape during unattended viewing was 61.24 (*SD* = 13.43) for convex, 63.41 (*SD* = 12.41) for concave and 123.82 (*SD* = 23.13) for 2D, whilst during attended viewing this was 60.00 (*SD* = 14.36) for convex, 60.44 (*SD* = 15.43) for concave and 118.24 (*SD* = 31.69) for 2D stimuli. The number of trials did not differ between viewing conditions for convex (*t*(33) = −079, *p* > .05), concave (*t*(33) = −1.71, *p* > .05) or 2D (*t*(33) = −1.62, *p* > .05) stimuli.

To investigate our hypotheses, namely that there is early processing related to shape from shading, and relatively later processing for shape from shading that is dependent on top-down attention, we used a series of repeated-measures analysis of variance (ANOVA). First, to test the effects of attention on the processing of each shape during each component, we conducted multiple attention (Unattended & Attended) × hemisphere (Left & Right) × shape (Concave, 2D & Convex) three-way ANOVAs with the amplitude of each ERP component as the dependant variable for each ANOVA. Further, separate hemisphere (Left & Right) × shape (Concave, 2D & Convex) ANOVAs were conducted with the amplitude of each ERP component as the dependant variable during unattended and attended conditions. By looking at differences in the ERPs for each shape in each viewing condition, we could examine if there are differences in neural activity related to the processing of 3D compared to 2D shape when attention is both guided and not guided to the stimuli. Additionally, by including hemisphere as an additional independent variable in our analysis, we could investigate the proposals of lateralisation that have been put forward for 3D shape processing (e.g., Taira et al., 2001; Gao et al., 2015; Séverac Cauquil et al., 2006). The Greenhouse-Geisser correction was applied to all analyses where the assumption of sphericity was violated. Further, all post-hoc comparisons were conducted using the Bonferroni correction to control for type 1 errors caused by multiple comparisons. All analyses were conducted using an alpha value of 0.05.

### Mass Univariate Analysis

To complement our traditional waveform analysis, we also carried out mass univariate analysis. This method uses a permutation approach to compare groups at each time-point across all electrodes whilst controlling for type 1 errors, providing the opportunity to observe the onset and offset of effects across multiple time points and electrodes. Further, this approach requires fewer assumptions to be made about the data due to the use of nonparametric tests and can aid in resolving issues that may arise from using small groups of electrodes for analysis that are thought to show a given component (Fields & Kuperberg, 2020; Groppe et al., 2011).

To conduct mass univariate analysis, we used the factorial mass univariate toolbox (Fields & Kuperberg, 2020), along with the mass univariate toolbox (Groppe et al., 2011) running in MATLAB. We used cluster-mass correction to control for type 1 errors, which uses a threshold value to group nearby electrodes and time-points to form clusters. The statistical values of these clusters are then summed to produce the cluster mass, and the significance of these clusters are then assessed by comparing the cluster mass to a null distribution produced from many thousands of permutations (Groppe et al., 2011; Pernet et al., 2015). Here, a cluster threshold of *p* ≤ .01 was used as this threshold has been found to better control type 1 error rates for focal components such as the P1 component (Fields & Kuperberg, 2020), and we defined neighbouring electrodes using a distance of 0.3759 in line with other research using similar electrode sets (e.g., Hudson et al., 2021; Itier & Durston, 2023). All analyses were conducted using 10000 permutations and an alpha value of *p* < .05.

We employed some strategies to improve the power of our mass univariate analysis. Before conducting the analysis, we decimated the data from 512 to 128 Hz to reduce the number of comparisons made (Fields & Kuperberg, 2020; Groppe et al., 2011; Luck, 2014). We also conducted analysis on a defined spatiotemporal region of interest (ROI) by only using a time-window where we expected an effect to occur and by limiting the number of electrodes in our analysis in accordance with where an effect was expected (Fields & Kuperberg, 2020; Luck, 2014). Specifically, we conduced analysis using an epoch of 100–350 ms (as each time point represented 8ms, the exact time range of the epoch was 104–354 ms), corresponding with the onset of the earliest component of interest (P1 component) and the offset of the latest component of interest (N2 component) identified by our collapsed localiser. We also limited analysis to posterior electrodes, given that depth cues have been found to modulate components at posterior sites (e.g., Gao et al., 2015; Mamassian et al., 2003; Omoto et al., 2010; Séverac Cauquil et al., 2006) and that predominantly posterior areas such as the intraparietal sulcus, inferior temporal gyrus and early visual cortex are involved in the processing of shape from shading (e.g., Georgieva et al., 2008; Gerardin et al., 2010; Taira et al., 2001). Therefore, we used the following 32 electrodes in our analysis: C1, C3, C5, T7, CP1, CP3, CP5, TP7, P1, P3, P5, P7, P9, PO3, PO7, O1 (Left hemisphere), C2, C4, C6, T8, CP2, CP4, CP6, TP8, P2, P6, P8, P10, PO4, PO8, O2 (Right hemisphere). This produced a total of 1056 comparisons. To examine our hypotheses, we first conducted a 3×2 (Shape × Attention) within-subjects mass ANOVA and then explored any main effects or interactions using a series of F-contrasts.

## Results

The current study investigated whether there is an early stage of shape from shading, and a relatively later stage of shape from shading that is reliant on top-down attention. Only the direction of significant results is described here.

Of the original cohort, eight participants were excluded from analysis as they reported being left-handed. This is because there is evidence that handedness can influence the hemispheric lateralisation of various cognitive processes (e.g., Johnstone et al., 2021; Knecht et al., 2000), including visuospatial attention (Hellige et al., 1994) and object perception (Willems et al., 2010), which may have led to a confound in the analysis of hemispheric effects in the current experiment. Moreover, there is evidence that the perception of shape from shading may also be affected by handedness due to variations in the direction of the light from above prior (Sun & Perona, 1998). A further two participants were excluded from analysis due to excessive artefacts in their EEG recording (e.g., excessive alpha oscillations), and another participant was excluded due to high trial rejection rates caused by excessive blinking. The final cohort consisted of 34 participants, with an age range between 18 and 29 years old (*M* = 20.32, *SD* = 2.69). There were 19 females, 12 males and 3 participants that did not conform to traditional gender norms. All participants were right-handed, and 11 participants (32.35% of the cohort) were bilingual. Further, 85% of participants possessed no neuropsychological conditions. Most participants reported perceiving the stimuli as intended at the end of the experiment, with 97.06% of participants reporting the intended perception of convexity, concavity and a flat (2D) stimulus. Further, as all participants reported perceiving at least 2 levels of depth in the stimuli (one participant did not perceive convexity, one participant did not perceive concavity and one participant did not perceive the ambiguous stimuli as 2D), no participants were excluded from the analysis. Moreover, accuracy for the colour search task was high in both passive (*M* = 97.05%, *SD* = 5.10) and active (*M* = 96.23%, *SD* = 5.55) viewing conditions, demonstrating participant engagement with the task.

### Unattended versus Attended Viewing

We conducted multiple attention (Unattended & Attended) × hemisphere (Left & Right) × shape (Concave, 2D & Convex) three-way ANOVAs, with the amplitude of each ERP component as the dependent variable. Difference waves for each shape, whereby waveforms from unattended viewing were subtracted from waveforms in attended viewing, were constructed to further explore any attention × shape interaction effects. The results of the ANOVAs are shown in Table 1, whilst a visual comparison of the ERPs from each condition can be found in the online Supplemental materials. The effect of attention on each component is discussed in turn.

**Table 1.** Showing the results of the three-way ANOVA for each component, comparing differences between unattended and attended conditions.

ANOVA effects	ERP components			
	P1	N1	P2	N2
Attention	*F* = 7.18	*F* = 0.24	*F* = 4.05	*F* = 10.99
	*p* = .01 *	*p* = .62	*p* = .05	*p* = .002 **
	*η2* = .179	*η2* = .007	*η2* = .109	*η2* = .250
Attention × hemisphere	*F* = 1.44	*F* = 4.87	*F* = 9.39	*F* = 0.74
	*p* = .23	*p* = .03 *	*p* = .004 **	*p* = .39
	*η2* = .042	*η2* = .129	*η2* = .221	*η2* = .022
Attention × shape	*F* = 0.13	*F* = 0.79	*F* = 1.19	*F* = 3.49
	*p* = .88	*p* = .45	*p* = .31	*p* = .03 *
	*η2* = .004	*η2* = .023	*η2* = .035	*η2* = .096
Attention × hemisphere × shape	*F* = 0.44	*F* = 1.71	*F* = 4.27	*F* = 0.72
	*p* = .59	*p* = .18	*p* = .01 *	*p* = .48
	*η2* = .013	*η2* = .049	*η2* = .115	*η2* = .021

*F* specifies test statistic; *p* indicates the significance value; *η2* represents effect size.

*Significant to *p* < .05, ** significant to *p* < .01, *** significant to *p* < .001.

#### P1 Component

The amplitude of the P1 component was larger during attended viewing (*M* = 1.19, *SD* = 1.69) compared to unattended viewing (*M* = 0.84, *SD* = 1.61), and this difference was significant (main effect of attention: *F* (1, 33) = 7.18, *p* = .011, *η2* = .179). However, the effects of attention on the P1 component were not greater for any specific shape, or in either hemisphere (*p* > .05 for all interaction effects).

#### N1 Component

Although a general effect of attention was not observed (*p* > .05 for main effect of attention) there was evidence to suggest that attention influenced the amplitude of the N1 component differently over each hemisphere (attention × hemisphere interaction: *F* (1, 33) = 4.87, *p* = .03, *η2* = .129). This reflected a decrease in N1 amplitude in attended (*M* = −0.61, *SD* = 2.44) compared to unattended (*M* = −0.89, *SD* = 2.70) viewing over the left hemisphere, whilst over the right hemisphere there was an increase in N1 amplitude in attended (*M* = −1.65, *SD* = 3.03) compared to unattended (*M* = −1.57, *SD* = 2.85) viewing. Despite this, follow-up t-tests did not reveal a significant difference in N1 amplitude between viewing conditions over the left (*t*(33) = 1.47, *p* = .15) or right (*t*(33) = −0.35, *p* = .72) hemisphere.

#### P2 Component

P2 amplitude in unattended viewing (*M* = 3.65, *SD* = 3.35) was greater than in attended viewing (*M* = 3.08, *SD* = 3.05), but this effect was at the threshold of significance (main effect of attention: *F* (1, 33) = 4.049, *p* = .05, *η2* = .109). There was however evidence that attention modulated the amplitude of the P2 component in a specific hemisphere (attention × hemisphere interaction: *F* (1, 33) = 9.39, *p* = .004, *η2* = .221). It was also indicated that there was an effect of lateralisation regarding the influence of attention on the P2 amplitude for a specific shape (three-way hemisphere × attention × shape interaction: *F* (2, 66) = 4.27, *p* = .01, *η2* = .115).

To explore this further, we conducted a 2 (attention) × 3 (shape) ANOVA with P2 amplitude separately over the left and right hemisphere. For the left hemisphere, this revealed that the P2 amplitude did not change between attentional conditions (main effect of attention: *F* (1, 33) = .91, *p* = .34, *η2* = .027) and there was no effect of attention on the sensitivity of the P2 component to shape (attention × shape interaction: *F* (1.68, 55.30) = 2.49, *p* = .10, *η2* = .070). However, in the right hemisphere the amplitude of the P2 component was significantly larger during unattended (*M* = 4.07, *SD* = 3.38) compared to attended (*M* = 3.21, *SD* = 3.21) viewing (main effect of attention: *F* (1, 33) = 7.80, *p* = .009, *η2* = .191). Nevertheless, we again found no evidence that attention modulated the sensitivity of the P2 component to different shapes (attention × shape interaction: *F* (2, 66) = 0.27, *p* = .76, *η2* = .008).

#### N2 Component

We observed larger N2 components during attended (*M* = 1.87, *SD* = 3.29) than unattended (*M* = 3.05, *SD* = 2.77) viewing (main effect of attention: *F* (1, 33) = 10.99, *p* = .002, *η2* = .250). Importantly, it appeared that attention influenced the amplitude of the N2 component to a greater extent for a specific shape (attention × shape interaction: *F* (2, 66) = 3.49, *p* = .03, *η2* = .096). Further analysis revealed that the increase in amplitude of the N2 component from unattended to attended viewing was greater for concave compared to 2D stimuli (*t*(33) = −2.13, *p* = .04) and convex stimuli (*t*(33), −2.29, *p* = .02), but there was no difference between convex and 2D stimuli (*t*(33) = −0.82, *p* = .41).

### Unattended Viewing

We hypothesised that there is early activity for shape from shading that is not dependant on attention, reflected by an increased amplitude in the P1 and N1 component for 3D compared to 2D stimuli. To investigate this the amplitude of each component was analysed using a hemisphere (Left & Right) × shape (Concave, Convex & 2D) ANOVA during the unattended condition. The results of this ANOVA for each component are shown in Table 2.

**Table 2.** Results of the 2×3 ANOVA for each component during the unattended condition.

ANOVA effects	ERP components			
	P1	N1	P2	N2
Shape	*F* = 0.38	*F* = 9.47	*F* = 0.15	*F* = 0.24
	*p* = .69	*p* < .001 ***	*p* = .32	*p* = .78
	*η2* = .011	*η2* = .223	*η2* = .034	*η2* = .007
Hemisphere	*F* = 3.80	*F* = 2.90	*F* = 8.31	*F* = 2.97
	*p* = .06	*p* = .09	*p* = .007 **	*p* = .09
	*η2* = .103	*η2* = .081	*η2* = .201	*η2* = .083
Shape × hemisphere	*F* = 3.02	*F* = 1.23	*F* = 1.38	*F* = 0.70
	*p* = .06	*p* = .30	*p* = .25	*p* = .50
	*η2* = .084	*η2* = .036	*η2* = .040	*η2* = .021

*F* specifies test statistic; *p* indicates the significance value; *η2* represents effect size.

*Significant to *p* < .05, ** significant to *p* < .01, *** significant to *p* < .001.

#### N1 Component

**Figure 3.**
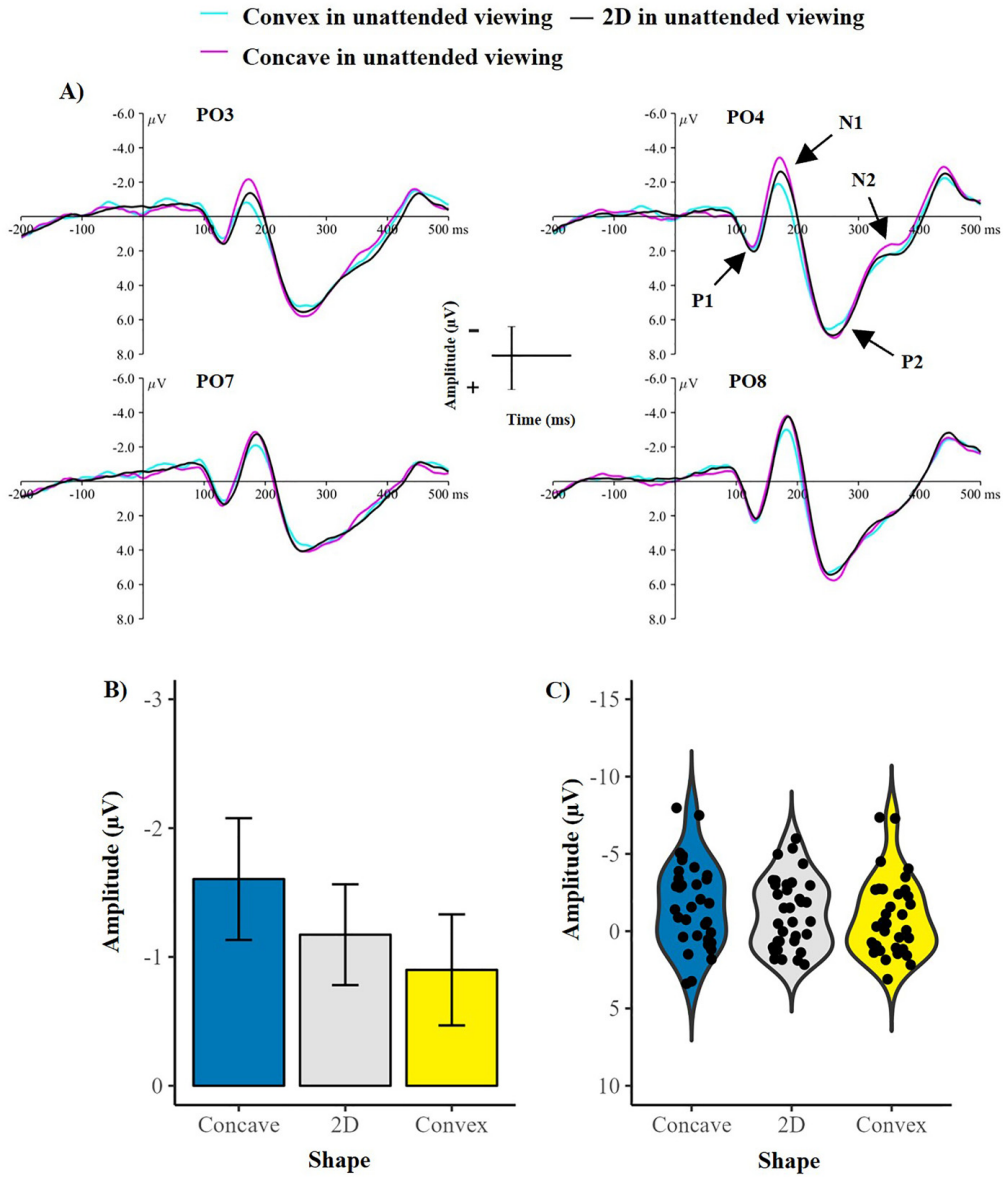
Demonstrating a greater N1 component for concave compared to 2D and convex shape during unattended viewing. (A) Showing grand-average ERPs for each shape during unattended viewing, with all ERP components labelled. (B) Demonstrating the mean amplitude of the N1 component during unattended viewing for each shape. Error bars represent the standard error of the mean. (C) violin plots showing the variation of the data for each shape, with each point representing a single participants’ mean N1 amplitude during unattended viewing for each shape. µV = microvolts, ms = milliseconds.

Results suggested that the amplitude of the N1 component was sensitive to changes in shape (main effect of shape: *F* (2, 66) = 9.47, *p* < .001, *η2* = .223), suggesting early processing of shape from shading. Figure 3 shows the grand-average ERPs from unattended viewing along with the mean amplitude and variation of the N1 component during unattended viewing across each shape. Follow-up comparisons revealed that concavity elicited a greater N1 amplitude than 2D (*t*(33) = −2.67, *p* = .01) and convex stimuli (*t*(33) = −4.45, *p* < .001), confirming early processing related to shape from shading when attention is not directed to the stimuli. The difference between convex and 2D stimuli was not significant (*t*(33) = −1.61, *p* = .11).

#### P2 Component

An asymmetry was only observed in the P2 component (main effect of hemisphere: *F* (1, 33) = 8.31, *p* = .007, *η2* = .201) with larger P2 amplitudes over the right (*M* = 4.07, *SD* = 3.38) than left (*M* = 3.22, *SD* = 3.28) hemisphere. No other effects were observed during unattended viewing of the stimuli (*p* > .05 for all other comparisons).

### Attended Viewing

Our second hypothesis was that there is later processing for shape from shading that occurs only when attention is guided to the stimuli, which functions to identify the 3D shape of the object. We predicted that this would be reflected by an increased amplitude of the P2 and N2 components for 3D compared to 2D stimuli. Therefore, we conducted analyses on the amplitude of each component during attended viewing in the same manner as for unattended viewing. The results of this ANOVA are shown in Table 3.

**Table 3.** Results of the 2×3 ANOVA for each component during the attended condition.

ANOVA effects	ERP components			
	P1	N1	P2	N2
Shape	*F* = 0.72	*F* = 12.93	*F* = 0.14	*F* = 6.35
	*p* = .49	*p* < .001 ***	*p* = .87	*p* = .003 **
	*η2* = .021	*η2* = .281	*η2* = .004	*η2* = .161
Hemisphere	*F* = 0.41	*F* = 5.22	*F* = 0.69	*F* = 4.51
	*p* = .40	*p* = .02 *	*p* = .41	*p* = .04 *
	*η2* = .021	*η2* = .136	*η2* = .021	*η2* = .120
Shape × hemisphere	*F* = 0.44	*F* = 5.58	*F* = 1.91	*F* = 0.22
	*p* = .59	*p* = .006 **	*p* = .15	*p* = .80
	*η2* = .013	*η2* = .145	*η2* = .055	*η2* = .007

*F* specifies test statistic; *p* indicates the significance value; *η2* represents effect size.

*Significant to *p* < .05, ** significant to *p* < .01, *** significant to *p* < .001.

#### N1 Component

We again found that the amplitude of the N1 component was sensitive to shape (main effect of shape: *F* (2, 66) = 12.93, *p* < .001, *η2* = .281), confirming early processing of shape from shading also when attention is deployed to the stimuli. There was an asymmetric distribution of the N1 component during attended viewing, with significantly greater amplitude N1 components over the right (*M* = −1.65, *SD* = 3.03) compared to left (*M* = −0.61, *SD* = 2.44) hemisphere (main effect of hemisphere: *F* (1, 33) = 5.22, *p* = .02, *η2* = .136). This was also accompanied by an effect of lateralisation (hemisphere × shape interaction: *F* (2, 66) = 5.58, *p* = .006, *η2* = .145), suggesting the difference in amplitude of the N1 component for concave compared to 2D and convex shape was greater over the right than the left hemisphere.

**Figure 4.**
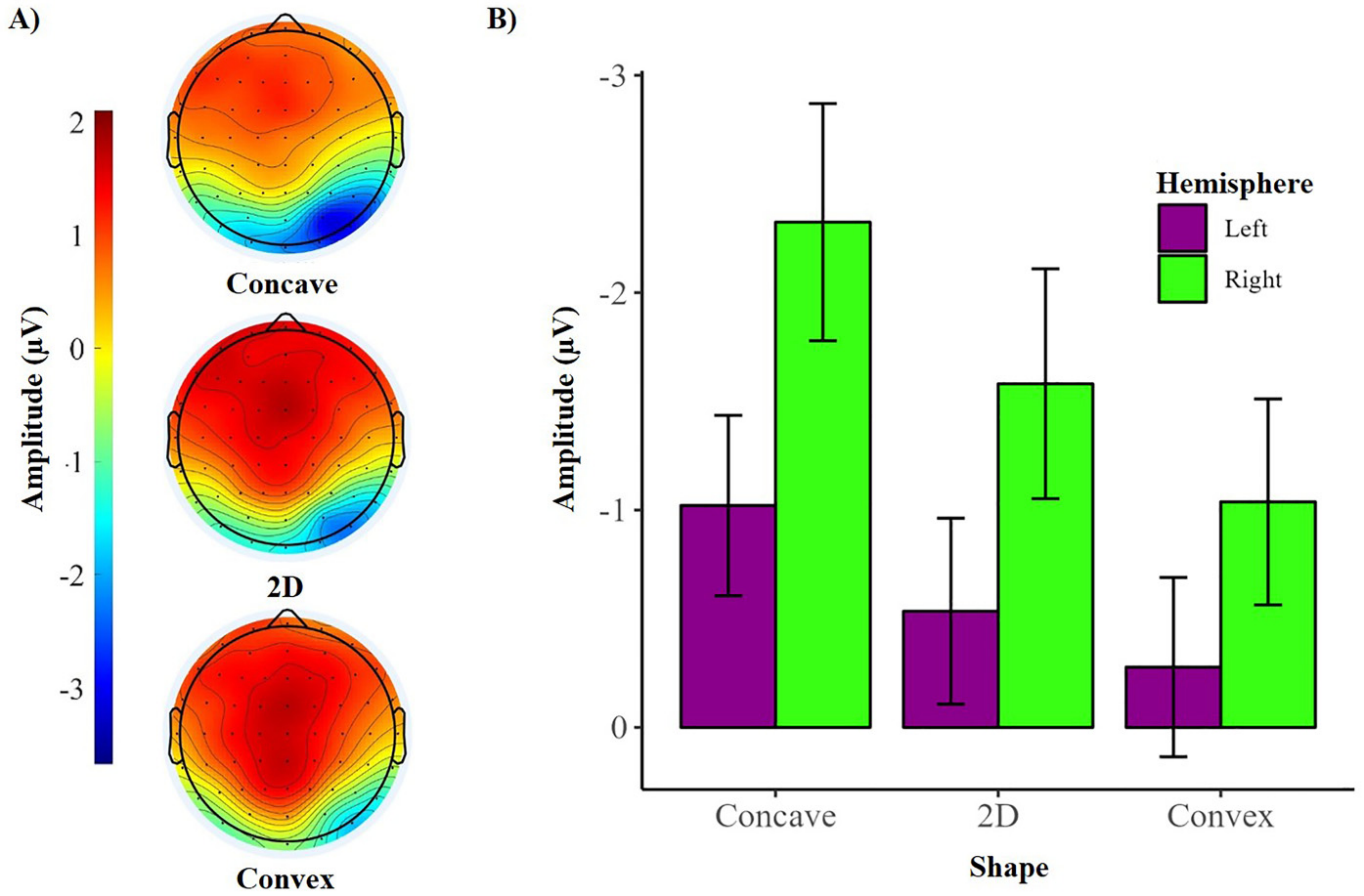
Showing an asymmetry of the N1 component during attended viewing, with greater N1 amplitudes in the right than left hemisphere. (A) Topographical distribution and mean amplitude of the N1 component during attended viewing. (B) Mean amplitude of the N1 component over each hemisphere for each shape. Error bars represent the standard error of the mean. µV = microvolts.

Figure 4 shows the asymmetry of the N1 component during attended viewing. A one-way ANOVA was conducted over each hemisphere to assess the effects of shape on the N1 amplitude. Over both, the left and right hemispheres, N1 amplitude differed in accordance with the shape presented (left: *F*(2, 66) = 6.47, *p* = .003, *η2* = .164; right: *F*(2, 66) = 16.68, *p* < .001, *η2* = .336). Specifically, N1 amplitude was greater for concave than 2D stimuli (left: *t*(33) = −2.65, *p* = .03; right: *t*(33) = −3.61, *p* = .003), and convex (left: *t*(33) = −3.46, *p* = .005; right: *t*(33) = −5.90, *p* < .001) stimuli. As these effects were greater over the right hemisphere, it suggests a dominance of the right hemisphere whilst attending to shape from shading. The comparisons between convex and the 2D stimulus were not significant (left: *t*(33) = −1.12, *p* = .81; right: *t*(33) = −2.22, *p* = .20).

#### N2 Component

**Figure 5.**
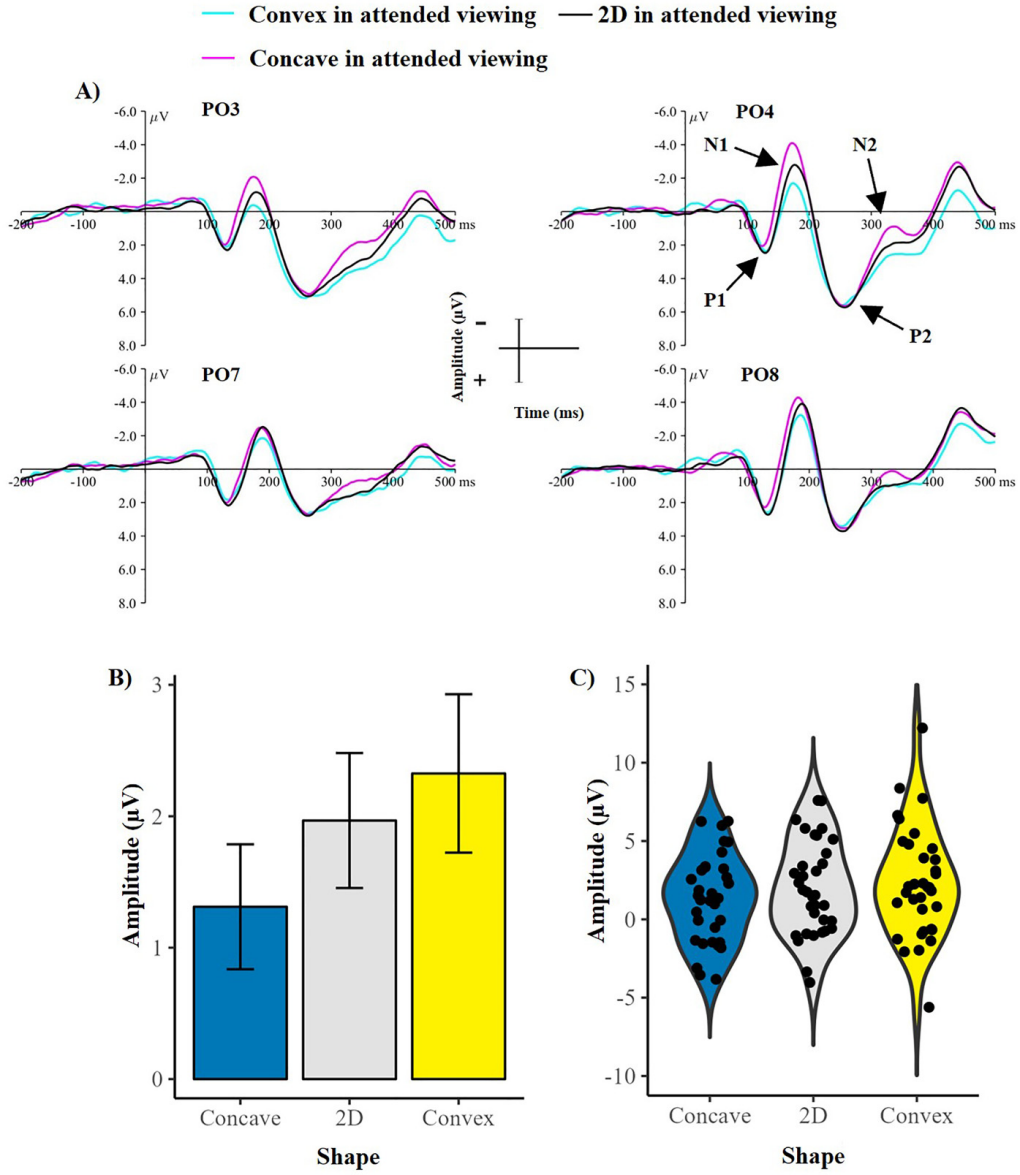
Demonstrating a greater N2 component for concave compared to 2D and Convex shape during attended viewing. (A) Showing grand-average ERPs for each shape during attended viewing, with all ERP components labelled. (B) Demonstrating the mean amplitude of the N2 component during attended viewing for each shape. Error bars represent the standard error of the mean. (C) Violin plots showing the variation of the data for each shape, with each point representing a single participants’ mean N2 amplitude during attended viewing for each shape. µV = microvolts, ms = milliseconds.

During attended viewing of the stimuli, the amplitude of the N2 component was significantly modulated by the shape of the stimuli (main effect of shape: *F* (2, 66) = 6.35, *p* = .003, *η2* = .161), suggesting a late processing stage in shape from shading that occurred only when attention is guided to the object. This effect is demonstrated by Figure 5. As was found for the N1 component, a greater amplitude N2 component was found for concave stimuli compared to 2D (*t*(33) = −2.84, *p* = .008) and convex (*t*(33) = −3.15, *p* = .003) stimuli, while the comparison between convex and 2D stimuli was not significant (*t*(33) = −1.17, *p* = .25). We also observed an asymmetry in the distribution of the N2 component, with larger amplitude N2 components over the right (*M* = 1.50, *SD* = 3.33) than left (*M* = 2.24, *SD* = 3.22) hemisphere (main effect of hemisphere: *F* (1, 33) = 4.51, *p* = .04, *η2* = .120). However, there was no significant effect of lateralisation (hemisphere × shape interaction: *F* (2, 66) = 0.22, *p* = .80, *η2* = .007).

### Mass Univariate Analysis

**Figure 6.**
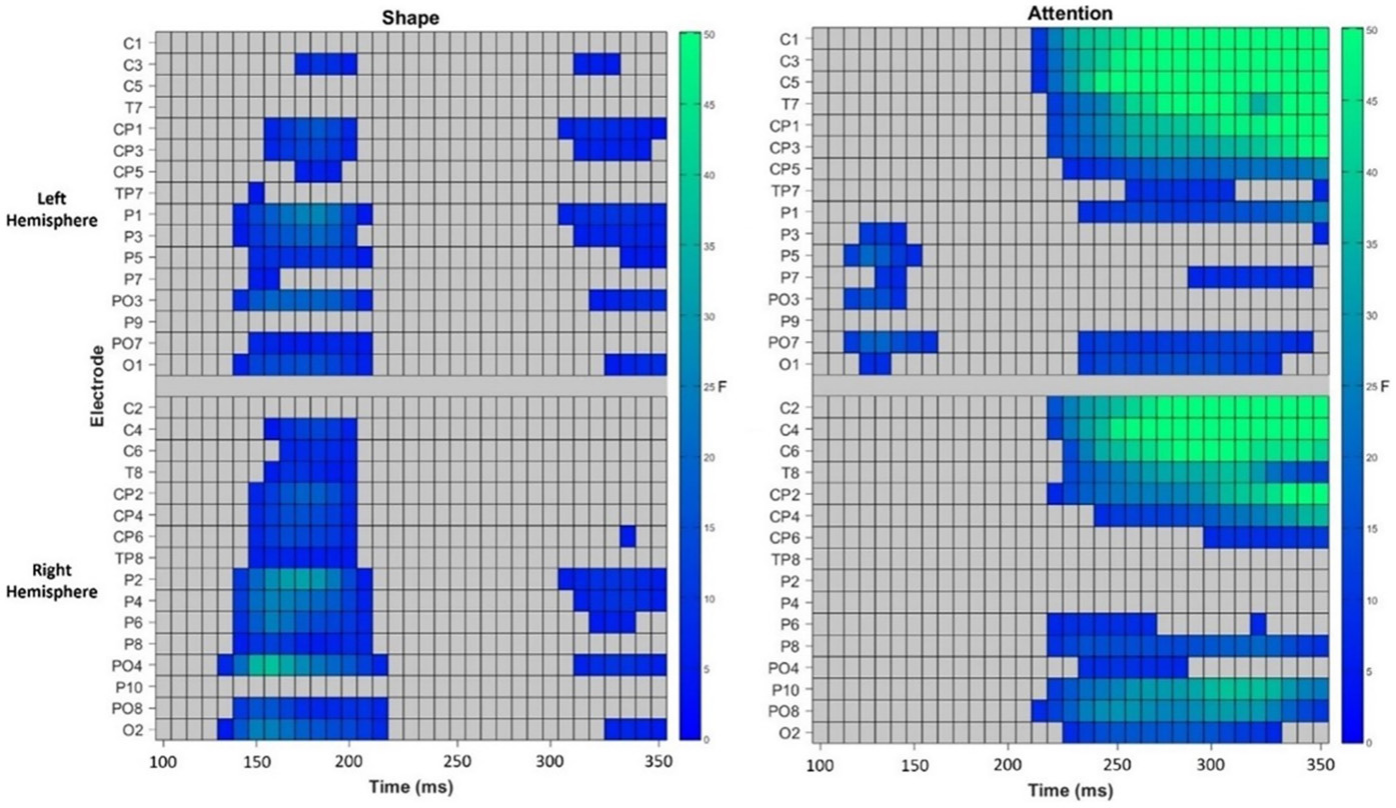
Raster plots showing a main effect of shape and attention at different latencies and electrodes. Left: Demonstrating two periods of significant differences for the main effect of shape. Right: Showing differences in waveforms between attentional conditions. ms = milliseconds, F specifies test statistic.

Figure 6 shows each time point at each electrode where the main effect of the ANOVA was significant. A main effect of shape was found with two periods of significant differences, which approximately aligned to the latency of the N1 and N2 components. For the first period of activity, the largest cluster in the right hemisphere was observed from 135–213 ms and peaked over PO4 at 158ms (*F* = 38.46, *p* < .001), but the largest cluster in the left hemisphere spanned from 143 ms until 205 ms and showed a maximum difference over electrode P1 at 182 ms (*F* = 26.59, *p* < .001). For the later activity, the largest clusters in both the left and right hemisphere began at 307 ms and lasted until 354 ms. The differences observed in both clusters peaked at 330 ms, although this was centred around electrode P1 in the left hemisphere (*F* = 10.35, *p* = .01) and electrode PO4 in the right hemisphere (*F* = 10.16, *p* = .02).

A main effect of attention was also observed at multiple time periods. An early band of significant differences was observed from 119 ms until 158 ms, which reflected a larger P1 amplitude in attended compared to unattended viewing. This effect was observed only in the left hemisphere, and the largest difference in the cluster was over P5 at 127ms (*F* = 20.59, *p* = .04). For the later band of differences, the largest clusters in both hemispheres lasted from 213 to 354 ms, which were largest over electrode C5 at 322 ms (*F* = 91.32, *p* < .001) in the left hemisphere, and at electrode C6 at 299 ms in the right hemisphere (*F* = 53.81, *p* < .001). These differences reflected a larger positivity for this latency range during the attended compared to unattended condition over more centrally located electrodes, whilst at the most posterior electrodes this effect was reversed, with larger negativities observed during attended compared to unattended viewing during this same period. Therefore, this demonstrates that our manipulation of attention effected the deployment of attention to each shape.

No significant interaction effect was observed for any electrodes at any time point (*p* > .05). However, we decided to conduct mass univariate analysis for each shape during each attentional condition, to explore our a priori hypothesis that top-down attention is necessary for relatively later stage processing for shape from shading.

**Figure 7.**
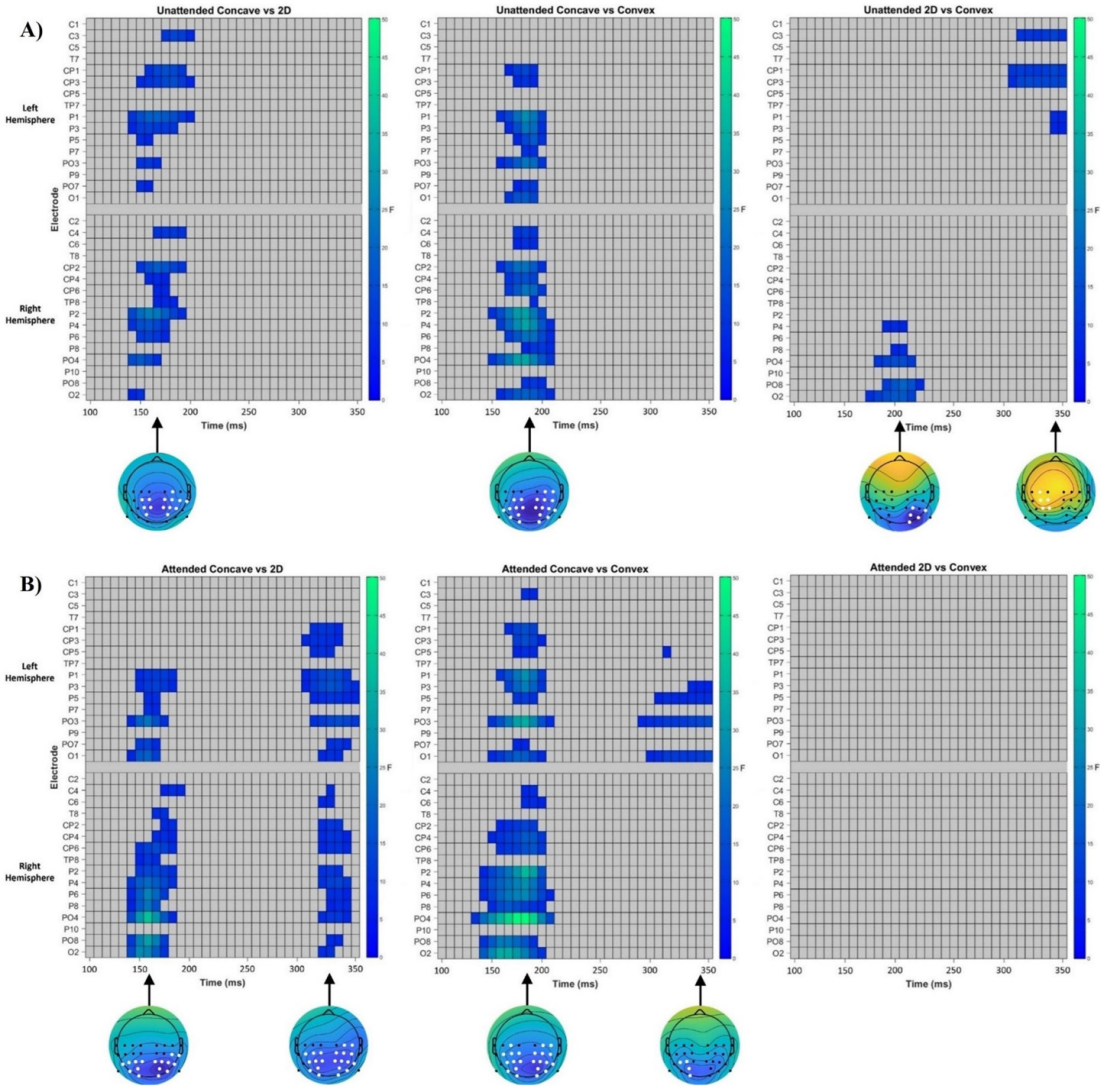
Raster plots showing early activity for concave shape during unattended viewing and later activity for concave shape during attended viewing only. (A) Significant time points and channels between each condition during unattended viewing. (B) Significant time points and channels between each condition during attended viewing. White points on topography maps indicate all significant electrodes at the indicated time point, whilst background colours on topography maps show the difference in voltage at that time point between the two conditions. ms = milliseconds, F specifies test statistic.

Significant differences between each shape during unattended and attended viewing are shown in Figure 7. During unattended viewing, the comparison of concave to 2D and to convex stimuli yielded a single band of significant differences that corresponded to the latency of the N1 component, supporting our finding of an early stage of processing for shape from shading. For concave compared to 2D stimuli, significant differences indicated a larger amplitude N1 component for concave stimuli. These differences were found in the left hemisphere from 143–197 ms, with a peak around electrode P1 at 166ms (*F* = 19.18, *p* = .01), and in the right hemisphere from 143 to 189 ms, with the largest difference observed at electrode P2 at 158 ms (*F* = 25.74, *p* = .01). Comparing concave and convex stimuli also revealed a larger N1 component for concave stimuli, with significant differences in the left hemisphere occurring from 158 to 197 ms, largest at electrode P1 at 182 ms (*F* = 28.81, *p* = .01), and in the left hemisphere from 150 to 205 ms, peaking at electrode PO4 at 182 ms (*F* = 33.27, *p* = .001).

Two small bands of differences were also observed during the comparison of 2D vs convex stimuli. The earlier band was isolated to the right hemisphere and represented a larger N1 component for 2D compared to convex stimuli, with significant differences at 174 ms until 221 ms, largest at 205 ms over electrode PO8 (*F* = 20.18, *p* = .04). Later differences were only observed over the left hemisphere where waveforms were more negative for convex compared to 2D stimuli during the period of 307–354 ms, with the greatest difference at 322 ms over electrode CP3 (*F* = 14.65, *p* = .03).

Two different periods of significant differences were found when comparing concave stimuli with 2D and convex stimuli during attended viewing, providing further evidence for a later processing stage in shape from shading that is dependent on top-down attention. For concave compared to 2D stimuli, this earlier band of differences again demonstrated a larger N1 component for concave stimuli. This difference was significant in the left hemisphere from 143–182 ms, peaking at 158 ms over electrode PO3 (*F* = 23.54, *p* = .03), although a larger difference between conditions appeared in the right hemisphere between 143–189 ms, largest at 158 ms over PO4 (*F* = 39.31, *p* = .003). The later band of differences corresponded with the latency range of the N2 component, with a greater amplitude N2 component for concave compared to 2D stimuli over the right hemisphere between 322–346 ms, peaking at 338 ms over electrode P4 (*F* = 13.92, *p* = .02). The temporal extent of differences over the left hemisphere was slightly longer, being 307–354 ms, greatest at electrode P3 at 330 ms (*F* = 16.74, *p* = .01).

A similar pattern was observed when comparing concave and convex stimuli. The earlier band of differences were significant over both hemispheres, representing a larger amplitude N1 component for concave compared to convex stimuli. Differences over the left hemisphere were significant between 150–205 ms, greatest at 182 ms over electrode PO3 (*F* = 34.81, *p* = .006). However, differences in the right hemisphere began slightly earlier, at 135–205 ms, and appeared to be larger than that in the left hemisphere, peaking at 182 ms over PO4 (*F* = 53.60, *p* < .001). The later band of differences were only observed over the left hemisphere occurring between 291–354 ms, greatest over PO3 at 354 ms (*F* = 15.16, *p* = .03), and represented a larger N2 component for concave compared to convex stimuli. No significant differences were observed over any electrodes at any time point when comparing 2D and convex stimuli (all *p* > .05).

#### An Effect of Attention or Practice?

Participants underwent 320 trials of the unattended condition before being instructed to attend to the 2D/3D stimuli. Therefore, it is possible that the effect that we attribute to attention could instead be a result of familiarity with the stimuli. Here, we test this by comparing the waveforms produced at the beginning of the unattended condition with those produced at the end of the unattended condition. We also compared the waveform at the end of the unattended condition to the waveform at the beginning of the attended condition. If the observed effect is due to attention, as we interpret, then the difference in the amplitude of the N1/N2 component for each shape should be similar between the first and last blocks of the unattended condition. However, the pattern of results should be different when the final blocks of the unattended condition are compared to the first blocks of the attended condition, showing that the attentional instructions and not time spent in the study are driving our results.

As such, we took the data from all 34 participants and averaged the ERPs produced from the first two blocks and last two blocks of the unattended condition, along with the first two blocks of the attended condition. The measurements of the N1 and N2 components are identical to that used in the main analysis. To investigate any familiarity effects, we ran a 3 (shape) × 2 (block) ANOVA for the N1 and N2 component separately. This was done first using the early and late blocks in the unattended condition, and then with the late blocks of the unattended condition and the early blocks of the attended condition.

#### Early versus Late Unattended Viewing

##### N1 Component

**Figure 8.**
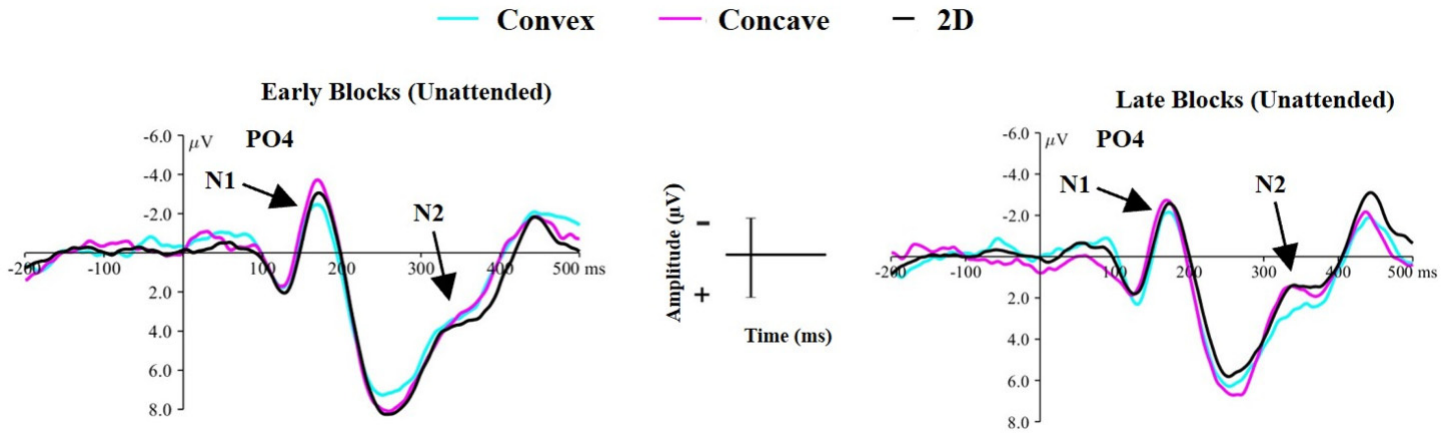
Showing that time spent in the unattended condition is not driving the observed effects in the N1/N2 components. Left: The waveform for each shape during the first two blocks of the unattended condition. Right: The waveform for each shape during the last two blocks of the unattended condition. µV = microvolts, ms = milliseconds.

The main effect of shape was not significant (*F*(2,66) = .39, *p* = .70, *η2* = .011), but the amplitude of the N1 component was significantly greater for early (*M* = −1.68, *SD* = 3.04) compared to later (*M* = −1.06, *SD* = 2.83) blocks of the unattended condition (main effect of block: *F*(1,33) = 6.44, *p* = .01, *η2* = .163). Furthermore, as shown by Figure 8 time spent in the unattended condition did not appear to mediate a difference in the amplitude of the N1 component for a given shape (non-significant shape × block interaction effect: *F*(2,66) = .27, *p* = .76, *η2* = .008).

##### N2 Component

There was no significant main effect of shape (*F*(2,66) = .37, *p* = .69, *η2* = .011), but there was a significant main effect of block (*F*(1,33) = 19.79, *p* < .001, *η2* = .375) with the amplitude of the N2 component becoming more negative between early blocks (*M* = 4.38, *SD* = 3.52) and later blocks (*M* = 2.55, *SD* = 3.55) of the unattended condition. Importantly, there was no significant shape × block interaction effect (*F*(2,66) = 1.17, *p* = .31, *η2* = .034), suggesting that time spent in the unattended condition did not cause a difference in the amplitude of the N2 component between convex, concave or 2D shape.

#### Late Unattended Viewing vs Early Attended Viewing

##### N1 Component

**Figure 9.**
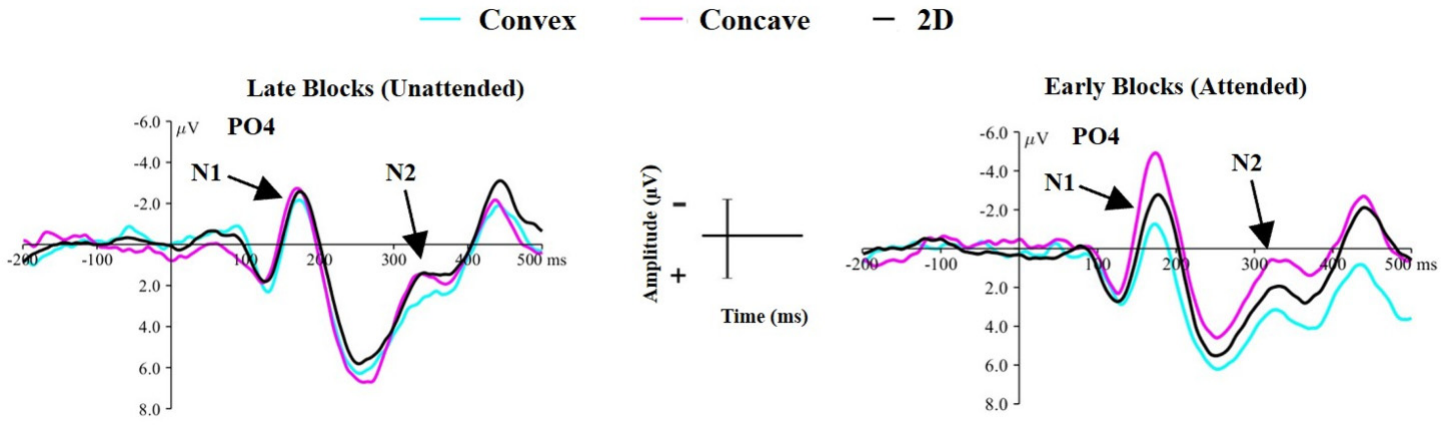
Demonstrating that attentional condition is driving the observed differences in the N1/N2 components. Left: the waveform for each shape during the last two blocks of the unattended condition. Right: the waveform for each shape during the first two blocks of the attended condition. µV = microvolts, ms = milliseconds.

The ANOVA produced a significant main effect of shape (*F*(2,66) = 6.13, *p* = .004, *η2* = .157), but there was no difference in the amplitude of the N1 component between blocks (*F*(1,33) = .24, *p* = .62, *η2* = .007). There was however a significant shape × block interaction effect (*F*(2,66) = 6.69, *p* = .002, *η2* = .169), indicating that a difference in N1 amplitude between shapes was mediated by attentional condition. This effect is shown in Figure 9.

One-way ANOVAs comparing the N1 amplitude produced from each shape in each block were conducted to explore this interaction effect. No significant difference in N1 amplitude was observed between any shape during the late blocks of the unattended condition (Main effect of shape: *F*(2,66) = .018, *p* = .982), but a significant difference in N1 amplitude was observed for early blocks of the attended condition (*F*(2,66) = 13.82, *p* < .001, *η2* = .295). Follow-up comparisons for early blocks of the attended condition revealed that concave stimuli elicited a greater amplitude N1 component than 2D (*t*(33) = −2.95, *p* = .006) and convex (*t*(33) = −4.69, *p* < .001) stimuli, whilst convex stimuli produced a significantly smaller N1 component than 2D stimuli (*t*(33) = −2.68, *p* = .01).

##### N2 Component

The ANOVA revealed a significant main effect of shape (*F*(2,66) = 4.40, *p* = .01, *η2* = .118), suggesting a significant difference in N2 amplitude elicited from the viewing of each shape. This was also accompanied by a significant main effect of block (*F*(1,33) = 4.39, *p* = .04, *η2* = .117) with significantly greater amplitude N2 component during the first blocks of the attended condition (*M* = 1.52, *SD* = 3.99) compared to the last blocks of the unattended condition (*M* = 2.55, *SD* = 3.55). Crucially, there was also a significant shape × block interaction effect (*F*(2,66) = 4.03, *p* = .02, *η2* = .109), suggesting that the manipulation of attention mediated a difference in the amplitude of the N2 component between shapes.

To explore this interaction effect, separate one-way ANOVAs were conducted for the amplitude of the N2 component in the late unattended blocks and the early attended blocks. For the late unattended blocks, there was no significant difference in N2 amplitude between any shape (main effect of shape: *F*(2,66) = 1.29, *p* = .28, *η2* = .037). However, in the early attended blocks a main effect of shape was found (*F*(2,66) = 7.19, *p* = .001, *η2* = .179) suggesting a difference in N2 amplitude elicited by the viewing of each shape. Follow-up comparisons revealed that concave stimuli elicited a larger N2 component compared to 2D (*t*(33) = −2.38, *p* = .02) and convex (*t*(33) = −3.46, *p* = .001) stimuli, but there was no significant difference between 2D and convex stimuli (*t*(33) = −1.69, *p* = .10). As such, differences in the N2 component elicited from viewing each shape was driven by the instruction to attend to the stimuli.

## Discussion

The current study aimed to test the hypothesis that there are two separate stages of processing for shape from shading; an earlier stage, to aid figure-ground segregation, and a relatively later stage of processing that is dependent on top-down attention to identify the 3D shape of the object. We used traditional waveform analysis along with mass univariate analysis to demonstrate processing related to shape from shading at around 150–200 ms post stimulus onset that occurred regardless of attentional condition. We also found that this processing was right lateralised only when participants were asked to attend to the objects. Interestingly, we found evidence for a relatively later stage of processing for shape from shading that occurred around 290–350 ms post stimulus onset. Our analysis also suggests that this stage of processing only occurred when top-down attention was deployed to the stimuli. Taken together, the data confirms our hypothesis that shape from shading consists of two stages, one early, pre-attentive process, and one late process that requires top-down attention. Finally, we found these results only with concave, rather than convex objects, with processing for concavity and convexity differing at both stages of processing. We discuss each of these main findings below.

We found early activity for shape from shading regardless of whether participants were guiding attention to the stimuli, reflected by a larger amplitude N1 component for concave compared to 2D shape. This is in line with previous research that has found depth cues to modulate the N1 component (e.g., Gao et al., 2015; Hou et al., 2006; Hideaki, 2018; Pegna et al., 2018; Séverac Cauquil et al., 2006). We suggest here that this activity is related to the initial processing of depth information, specifically functioning to segregate the object from the background. This is consistent with the body of work that demonstrates that depth cues are important for figure-ground segregation (Bertamini & Wagemans, 2013; Kanizsa & Gerbino, 1976; Kimchi & Peterson, 2008; Salvagio et al., 2008) and the suggestion that early ERPs occurring before 200ms, such as the P1 and N170, may reflect figure-ground segregation (Schendan & Lucia, 2010; Schendan & Ganis, 2015). There is also evidence to suggest that figure-ground segregation occurs, at least in part, without the need for top-down attention. For instance, it has been argued that boundary detection for figure-ground segregation occurs independently of visual attention (Poort et al., 2012), and changes in figure-ground organisation whilst participants attended to an unrelated target judgement task can lead to congruency effects in the judgement task even when participants reported being unaware of the figure-ground organisation (Kimchi & Peterson, 2008). Therefore, we suggest that the differences in early activity during unattended viewing of our stimuli, observed in the N1 component, represent the rapid extraction of depth from shading for use in processes such as figure-ground segregation.

When participants were asked to actively attend to the stimuli, we found that the magnitude of this N1 effect for concave shape was greater in the right than the left hemisphere. This right lateralisation effect is consistent with previous findings that suggest a right hemispheric dominance in the processing of depth perception (Baecke et al., 2009; Carmon & Bechtoldt, 1969; Durnford & Kimura, 1971; Iwami et al., 2002; Nishida et al., 2001; Taira et al., 2001), and ERP studies that have demonstrated an enhanced N1 component for depth perception in the right hemisphere (Gao et al., 2015; Séverac Cauquil et al., 2006; Séverac Cauquil et al., 2009; Yue et al., 2020). The right hemispheric lateralisation of our N1 component may have originated in parietal areas, given evidence that the right IPS was activated when participants attended to shape from shading stimuli (Taira et al., 2001), and source localisation that indicated a parietal source for a right lateralised N1 component elicited by depth cues (Séverac Cauquil et al., 2006). Given the role of the IPS in top-down attention (e.g., Corbetta & Shulman, 2002; Hahn et al., 2006; Moos et al., 2012), we suggest that the effect of attention observed on our N1 component reflects the selection of depth cues for further processing. This is in line with research demonstrating that the N1 component represents object-selective attention (Martínez et al., 2006; Martínez et al., 2007; Valdes-Sosa et al., 1998) and work suggesting that the N1 component reflects attention to depth information (Gao et al., 2015; Kasai & Morotomi, 2001). It is also unlikely that this effect is due to participants only attending to concave stimuli, as an increased P1 amplitude was found in attended compared to unattended viewing for all stimuli, in line with evidence demonstrating that attention modulates the amplitude of the P1 component (Gonzalez et al., 1994; Luck et al., 1990; Slagter et al., 2016; for a review see Luck & Kappenman, 2012). Therefore, the right lateralisation of the N1 component for concavity reinforces the notion that some representation of depth information is available to the attentional system at an early stage in processing.

In the current study, we also found a larger N2 component between 290–350ms for concave compared to 2D stimuli only whilst participants attended to the stimuli. Previous studies have suggested that the posterior N2 component is related to processes involving shape processing (Bowden et al., 2015; Proverbio et al., 2004) and other research has also related the N2 component to the processing of 3D shape (Gao et al., 2015; Mamassian et al., 2003; Pegna et al., 2018). For instance, when observers were presented with multiple orientations of a shape from shading stimuli, an N2 component around 300ms post stimulus onset appeared to correspond with the change of 3D shape that occurred in different stimulus orientations (Mamassian et al., 2003). The authors therefore suggested that this N2 component reflected 3D shape processing for shape from shading. Other evidence to suggest that the N2 component reflects the processing of 3D shape comes from Gao et al., (2015). Here, the authors recorded ERPs whilst participants viewed planar graphics with no depth or shape information, perspective drawings containing depth but no shape information, or a 3D object containing both depth and 3D shape information. They found that 3D objects produced a larger N2 component than perspective drawings over the left hemisphere, suggesting that the N2 component reflects processing related to 3D shape. Such a notion is also supported by research that has found the N1 component to be sensitive to global 3D shape, whilst detailed processing of local shape structure appeared to occur in the latency of the N2 component (Leek et al., 2016). This supports our suggestion that initial rapid processing of 3D information (e.g., depth from shading) is represented by the N1 component, whilst the increased N2 amplitude for concave stimuli in our experiment reflects detailed processing of the object that is necessary for the recovery of 3D shape information.

An interesting finding here is that we found early and late stages of processing related to concave, but not convex shape from shading. In fact, we found differences in activity for the processing of each 3D shape at both stages of processing. Such a finding may not be surprising given previous work. For instance, in a feature search task conducted by Sun and Perona (1996) participants reported perceiving the shape of convex, but not concave distractors. This is interesting, as it suggests that concavities, but not convexities, require guided attention to be perceived. Moreover, previous research has suggested that concavities play a unique role in shape processing (Barenholtz et al., 2003; Leek et al., 2012; Lim & Leek, 2012), such as facilitating object processing through part segmentation (Biederman, 1987; Hoffman & Richards, 1984). Finally, the anterior portions of the lateral occipital complex (LOC) have been shown to be more sensitive to convex than concave shape (Haushofer et al., 2008), suggesting that different neural processes may be responsible for supporting the perception of convexity and concavity. We suggest that in the context of shape from shading, such differences may be explained by a convexity prior, whereby observers are more likely to assume that an ambiguous shaded object is convex than concave (Kleffner & Ramachandran, 1992; Langer & Bülthoff, 2001; Liu & Todd, 2004; Todorović, 2014). Such a convexity prior may operate through feedback activity, perhaps from areas involved in 3D shape processing as proposed by Gerardin et al. (2010). Therefore, prediction error signals to overcome this convexity prior may be required to perceive concavity, which could manifest as increased feedforward signal within the same cortical network (Hardstone et al., 2021). Given that attention has also been suggested to facilitate such prediction error signals (for a review see den Ouden et al., 2012), our effects of attention for concavity in this study may also be in part due to attentional enhancement that was necessary to create a prediction error signal to overcome the convexity prior and perceive concave shape. Due to the speculative nature of such a suggestion, further investigation into how feedback and feedforward activity in cortical networks support the perception of 3D shape would be highly beneficial to help develop our understanding of the differences in processing that underlie the perception of convexity and concavity.

It could be argued that our differences between concavity and convexity may be due to participants not perceiving the snowflake stimulus as convex at 0°, especially considering that participants did not make explicit responses to the 3D or 2D stimuli over the course of our experiment. However, it is worth noting that almost all participants in our study reported perceiving the stimuli as intended at the end of the experiment. Furthermore, identical stimuli were used in multiple experiments by Sapir et al. (2021), with these stimuli being consistently being reported as convex and concave at orientations of 0° and 180°, respectively. We also confirmed that these stimuli were perceived as intended in a pilot study conducted prior to the experiment presented here. Therefore, it seems unlikely that the differences observed in our ERPs are due to issues in the perception of the stimuli. Although we demonstrated that the modulation of the ERPs is due to the instruction to attend to the stimuli, a possibility that needs to be considered is whether our results are due to attention, or due to additional information being given about the stimulus of interest. That is, being given information detailing how the object should be perceived may be responsible for the changes in ERPs observed. Despite clear effects of attention on the ERP waveforms in the current study, this is a limitation that should be addressed in future studies.

In the current study, we provide evidence that shape from shading consists of two distinct processes, an early process that occurs without the need for guided attention and a relatively later stage of processing that only occurs when top-down attention is deployed to the object. We suggest that the early processing observed in the N1 component likely reflects rapid processing of depth from shading for use in processes such as figure-ground segregation. Later activity, as seen in the N2 component, may reflect the recovery of 3D shape from shading, which is reliant on the deployment of top-down attention to the object. We also found these results with concave, but not convex objects. As such, our findings indicate that the recovery of concave shape from shading is reliant on the deployment of top-down attention.

## Supplemental Material

sj-docx-1-ipe-10.1177_20416695251350000 - Supplemental material for Three-dimensional shape from shading is modulated by top-down attention: Evidence from event-related potentials Supplemental material, sj-docx-1-ipe-10.1177_20416695251350000 for Three-dimensional shape from shading is modulated by top-down attention: Evidence from event-related potentials by Joshua P. Matthews, Debra L. Mills, and Ayelet Sapir in i-Perception

## Notes

1. A pilot study (*n* = 17) using a forced choice paradigm (only convex or concave responses) was conducted to ensure participants did not perceive the 2D stimulus as a reliable 3D shape. Shape judgements for the 3D shape were significantly more consistent than for the 2D shape (*t*(16) = −4.33, *p* < .001).

2. We tested the suitability of using a 200ms display time for the 3D stimuli in a separate pilot experiment (*n* = 31). We found no significant differences between the consistency of shape judgments when using 400, 300 and 200 ms display times (*F*(2,56) = 2.31, *p* = .10, *η2* = .076).
