# Supplementary material for: Three-dimensional shape from shading is modulated by top-down attention: Evidence from event-related potentials
Source: Iperception. 2025 Jul 13;16(4):20416695251350000. doi: 10.1177/20416695251350000 (PMC12260317; doi:10.1177/20416695251350000)
Supplement: sj-docx-1-ipe-10.1177_20416695251350000 - Supplemental material for Three-dimensional shape from shading is modulated by top-down attention: Evidence from event-related potentials [file sj-docx-1-ipe-10.1177_20416695251350000.docx]

**Supplementary Materials**

Figure showing the difference in ERP waveforms between unattended and attended viewing


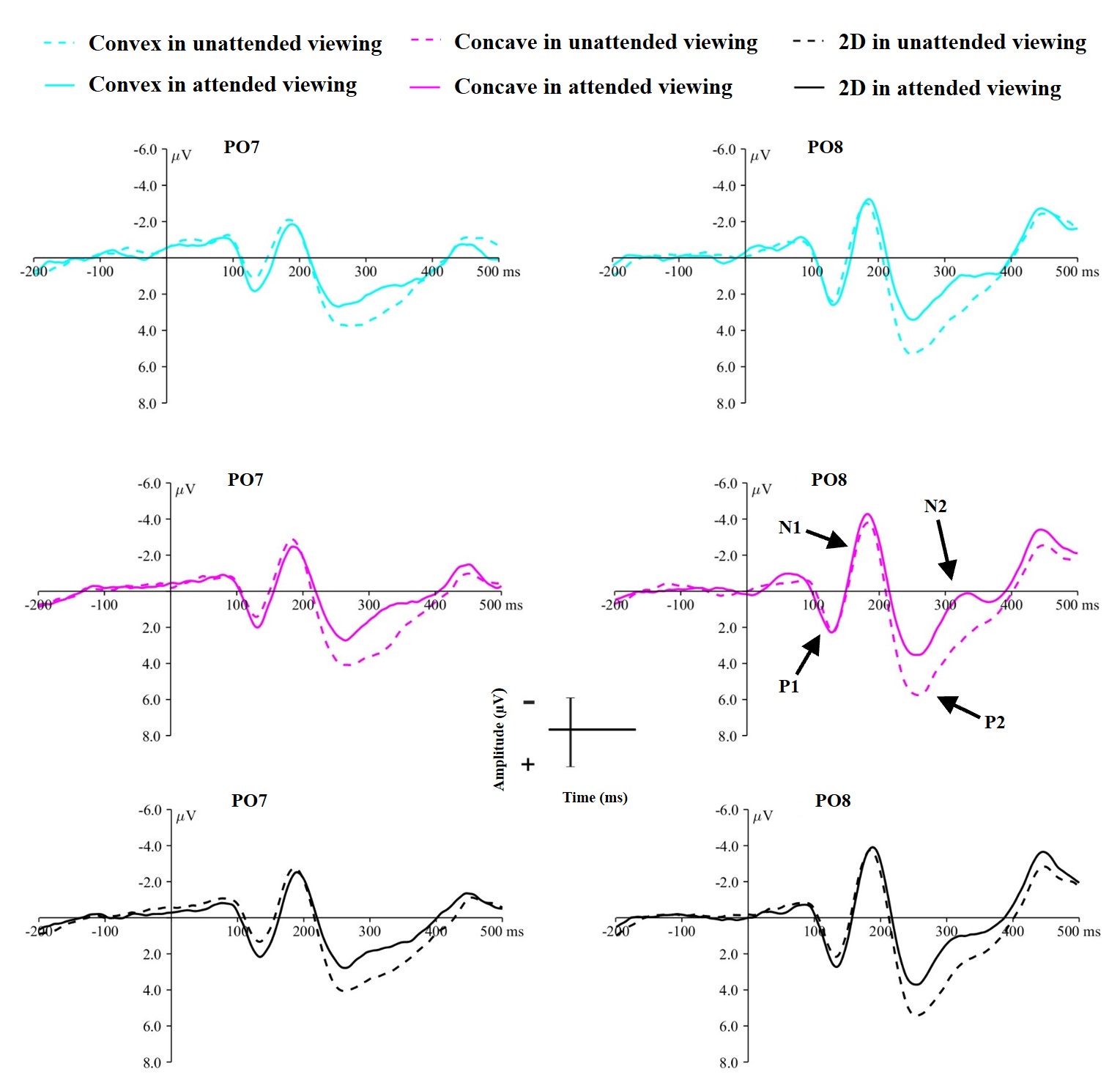


**Figure 10.** Showing the differences in waveforms between unattended and attended viewing for each component and shape. *ms* = milliseconds, *µV* = microvolts.
